# Visual adaptation alters the apparent speed of real-world actions

**DOI:** 10.1038/s41598-017-06841-5

**Published:** 2017-07-27

**Authors:** George Mather, Rebecca J. Sharman, Todd Parsons

**Affiliations:** 1 University of Lincoln, School of Psychology, Lincoln, LN5 7AY UK; 2 University of Stirling, Psychology, Stirling, FK9 4LA UK

## Abstract

The apparent physical speed of an object in the field of view remains constant despite variations in retinal velocity due to viewing conditions (velocity constancy). For example, people and cars appear to move across the field of view at the same objective speed regardless of distance. In this study a series of experiments investigated the visual processes underpinning judgements of objective speed using an adaptation paradigm and video recordings of natural human locomotion. Viewing a video played in slow-motion for 30 seconds caused participants to perceive subsequently viewed clips played at standard speed as too fast, so playback had to be slowed down in order for it to appear natural; conversely after viewing fast-forward videos for 30 seconds, playback had to be speeded up in order to appear natural. The perceived speed of locomotion shifted towards the speed depicted in the adapting video (‘re-normalisation’). Results were qualitatively different from those obtained in previously reported studies of retinal velocity adaptation. Adapting videos that were scrambled to remove recognizable human figures or coherent motion caused significant, though smaller shifts in apparent locomotion speed, indicating that both low-level and high-level visual properties of the adapting stimulus contributed to the changes in apparent speed.

## Introduction

A great deal is known about how the visual system of the brain responds to stimuli received by the eye. The bulk of this research has used artificial stimulus sets such as sine-wave gratings, which allow precise parametric control of the visual properties driving lower level processing in striate and extrastriate cortex. For example, early cortical areas are known to contain neurons that respond selectively to the local retinal orientation and direction of simple visual patterns^1^. However the visual system evolved to process images depicting more complex natural scenes, and neural circuits at higher levels of analysis in the cortex may be largely unresponsive to these artificial stimulus sets^2–4^. For example, the perceived stability of visual properties such as size, shape, lightness and colour (the perceptual constancies) cannot be explained solely by responses in early visual areas that vary with retinal image parameters^5, 6^, but may require high-level processes operating over extended areas of the visual field, involving large ensembles of neurons^7^.

In motion perception, the apparent speed of an object in the field of view remains constant despite variations in retinal velocity due to viewing conditions. For example, visual objects such as people and cars appear to move at the same objective speed regardless of viewing distance (velocity constancy^8, 9^). Some researchers have viewed velocity constancy as an extension of size constancy, while others have suggested that the temporal dynamics of the image are important for maintaining velocity constancy^8–11^. Little is known about how the responses of neurons in early visual areas of the cortex contribute to velocity constancy. The present experiments addressed this issue using a novel motion adaptation paradigm in which participants judged the speed of a common real-world action, human locomotion, after exposure to different kinds of adapting pattern. The speed of human locomotion was selected for study because it is particularly important for social interactions and is known to support subtle judgements of meaning, emotion and intent^12–17^. The first two experiments show that prior viewing of speeded-up or slowed-down video recordings of locomotion causes changes in the perceived speed of locomotion in subsequently viewed video clips^18^. Later experiments investigate whether this adaptation effect can be explained in terms of known changes in the responsiveness of low-level neurons, or implicates higher-level processes involved in velocity constancy^19^. We tested whether adaptation depends on playback speed per se or on retinal speed, and then investigated whether image flicker plays a role. Although retinal stimulus parameters were found to be important, results were qualitatively different from those obtained in previously reported studies of low-level retinal velocity adaptation, and indicated that image temporal frequency properties contribute to maintaining speed constancy in perception.

## Results

### Experiments 1 and 2: Adaptation to walking and running

In each test trial experimental participants viewed a short video excerpt taken from a recording of people walking along a local High Street, or running in a sports event (London Marathon). The videos contained moving figures at a range of distances, speeds and directions, as is typical in everyday scenes. They were shown at playback speeds ranging from slow-motion (0.48x) to fast-forward (1.44x) relative to standard playback speed (1x, which represents real-life speed). After viewing each clip the participant made a binary judgement as to whether the action in the clip appeared to be performed at a slower or faster pace than natural pace. From the pattern of responses to different test speeds we were able to estimate the playback speed which was judged as natural by participants (full details of experimental procedures are given in Methods).

**Figure 1:**
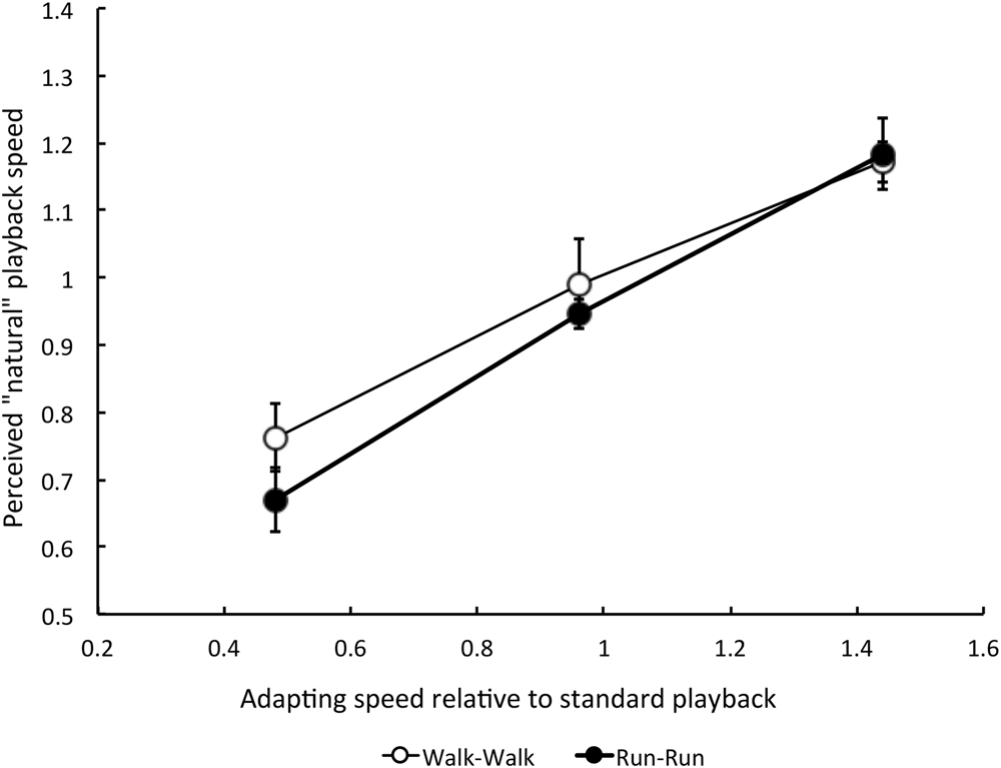
Results of Experiments 1 and 2, showing the playback speed at which locomotion appeared natural as a function of the adapting speed relative to standard playback. *Open circles* Data from Experiment 1 (walkers). *Filled circles* Data from Experiment 2 (runners). Values below 1.0 indicate that playback speed had to be slower than standard (1x) in order to appear natural. Values above 1.0 indicate that playback speed had to be faster than standard to appear natural. Each data point represents the mean 50% point (P50) of five participants (+/−1 SE) derived from best-fitting cumulative normal distributions applied to each participant’s response rates.

We found that viewing of a slow-motion (SM) video for 30 seconds caused participants to perceive subsequently viewed clips played at standard speed as too fast, so playback had to be slowed down in order to appear natural. Conversely, after viewing fast-forward (FF) videos for 30 seconds, playback speed had to be increased in order to appear natural. However, adaptation to standard speed itself (SS; actually 0.96x playback on our equipment) did not affect apparent locomotion speed. Figure 1 plots the mean playback speed that appeared natural in each of the three adapting speed conditions; using the walking videos (Experiment 1: Walk-Walk; unfilled circles) and the running videos (Experiment 2: Run-Run; filled circles). The perceived speed of locomotion in the test videos shifted towards the speed of the adapting video, as though perceived speed normalises to recently viewed speeds (‘re-normalisation’). Repeated Measures Analysis of Variance tests applied to the results of each experiment confirmed a significant effect of adapting speed in both (Expt. 1: F (2, 8) = 23.43, p = 0.0001; Expt. 2: F (2, 8) = 25.99, 0.0001; n = 5).

### Experiment 3: Cross-adaptation between walking and running

At SS playback, the mean retinal speed of locomotion in the walking videos was roughly half that in the running videos (1.76°/sec and 3.06°/sec respectively), though both videos contained figures moving at a range of retinal speeds (see Methods for a description of how these speeds were calculated). Manipulations of playback speed shifted these speed distributions to lower or higher retinal speeds. The data shown in Fig. 1 do not allow us to determine whether the adaptation is driven by adapting speed relative to a norm value for natural speed (SS playback for both walking and running), or by the retinal speed of the adapting stimuli.

To distinguish between these two alternatives, Experiment 3 measured cross-adaptation: participants adapted to walking videos and were tested using running videos (Walk-Run) or vice-versa (Run-Walk), again reporting whether the locomotion in test videos appeared to be faster or slower than a natural speed. If adaptation is driven by norm-based speed, then shifts in perceived speed should be equal in the two cross-adaptation conditions because the same norm-based adapting speeds were used in both, and results were similar in Experiments 1 and 2. If adaptation is driven by retinal speed, then results should differ between the conditions because adaptation in Run-Walk involves much higher retinal speeds than those in Walk-Run.

**Figure 2:**
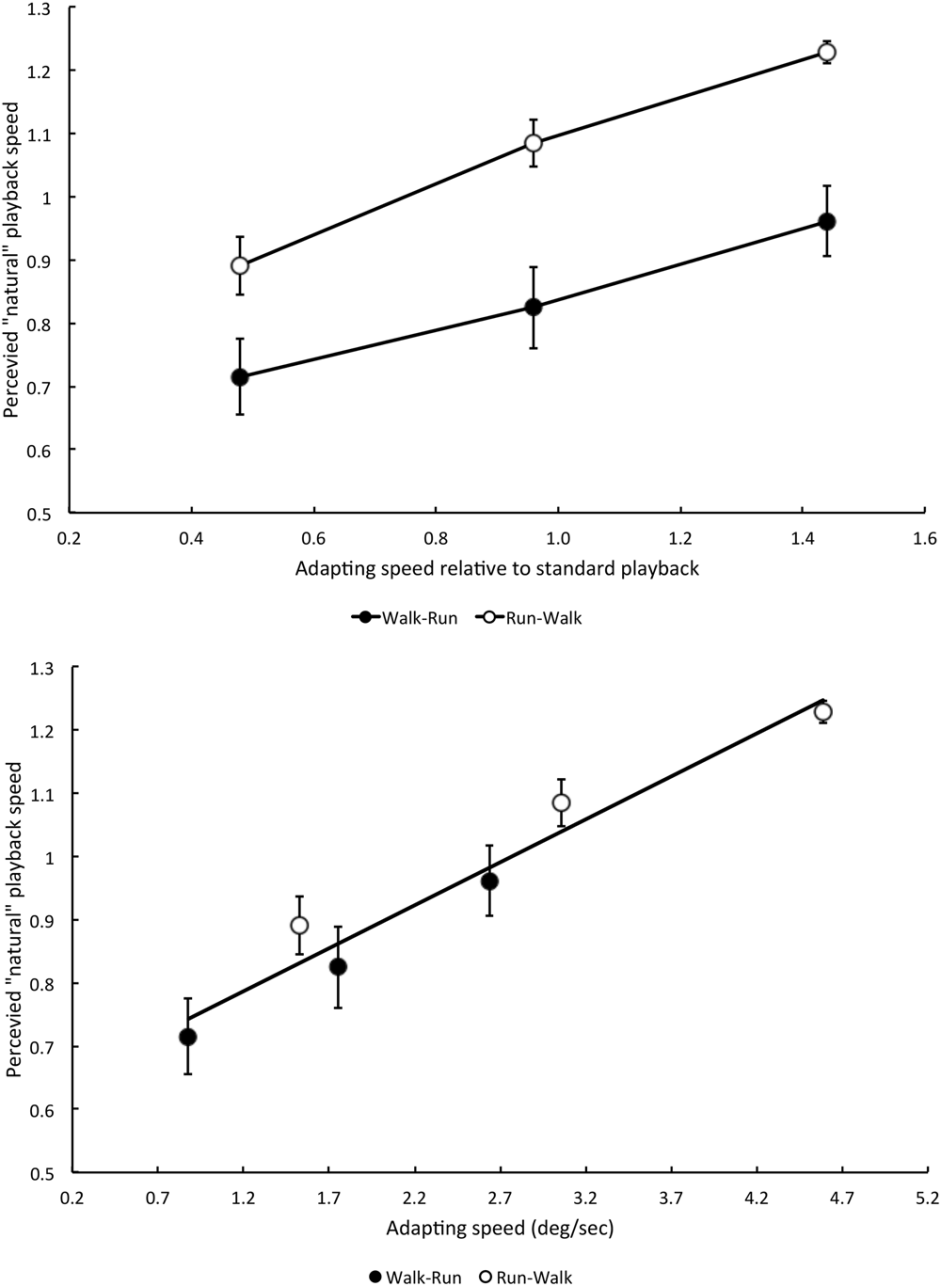
Results of Experiment 3 involving cross-adaptation between running and walking. *Filled circles* Data using walking adapt and running test videos (Walk-Run). *Unfilled Circles* Data using running adapt and walking test videos (Run-Walk). The upper graph plots results in terms of the norm-based speed of the adapting pattern (i.e. relative to standard-speed playback). The lower graph plots results in terms of the mean retinal speed of the adapting pattern. Figure convention as in Fig. 1. Each data point represents the mean P50 of five participants (+/−1 SE).

Results are shown in Fig. 2. Filled circles represent data from the Walk-Run condition, and unfilled circles represent data from the Run-Walk condition. The top graph shows results in terms of norm-based adapting speed (i.e. relative to standard-speed playback). Walk-Run data show adaptation-induced reductions in apparent locomotion speed, whereas Run-Walk data mostly show increases in apparent locomotion speed. The difference between the two cross-adaptation conditions is significant according to a Repeated Measures Analysis of Variance (F (1, 4) = 11.85, p = 0.0262, n = 5), as is the main effect of adapting speed (F (2, 8) = 40.63, p = 0.0001, n = 5). The interaction between cross-adaptation and speed was not significant (F (2, 8) = 1.54, p = 0.272, n = 5).

When results are plotted in terms of the retinal speed of the adapting pattern (lower graph) the results of the two conditions fall into alignment close to a single linear function (Pearson r^2^ = 0.95).

### Experiment 4: Adaptation to row-scrambled stimuli

The results of the previous experiment are consistent with the hypothesis that changes in apparent locomotion speed are driven by the retinal speed of the adapting stimulus regardless of whether it depicts walking or running, indicative of low-level adaptation. In Experiment 4 we tested whether the presence of recognisable human forms during adaptation is important for changes in perceived locomotion speed. We repeated the Run-Run condition of Experiment 1, but spatially scrambled the adapting stimulus to destroy form cues in the running figures: The rows of pixels in each frame of the adapting video were randomly shuffled. The same shuffled order was used in all frames of a given adaptation session (to preserve the horizontal motion signals within each row), but different scrambled row orders were used in different presentations (see Supplementary Video V1 and Supplementary Figure F1).

**Figure 3:**
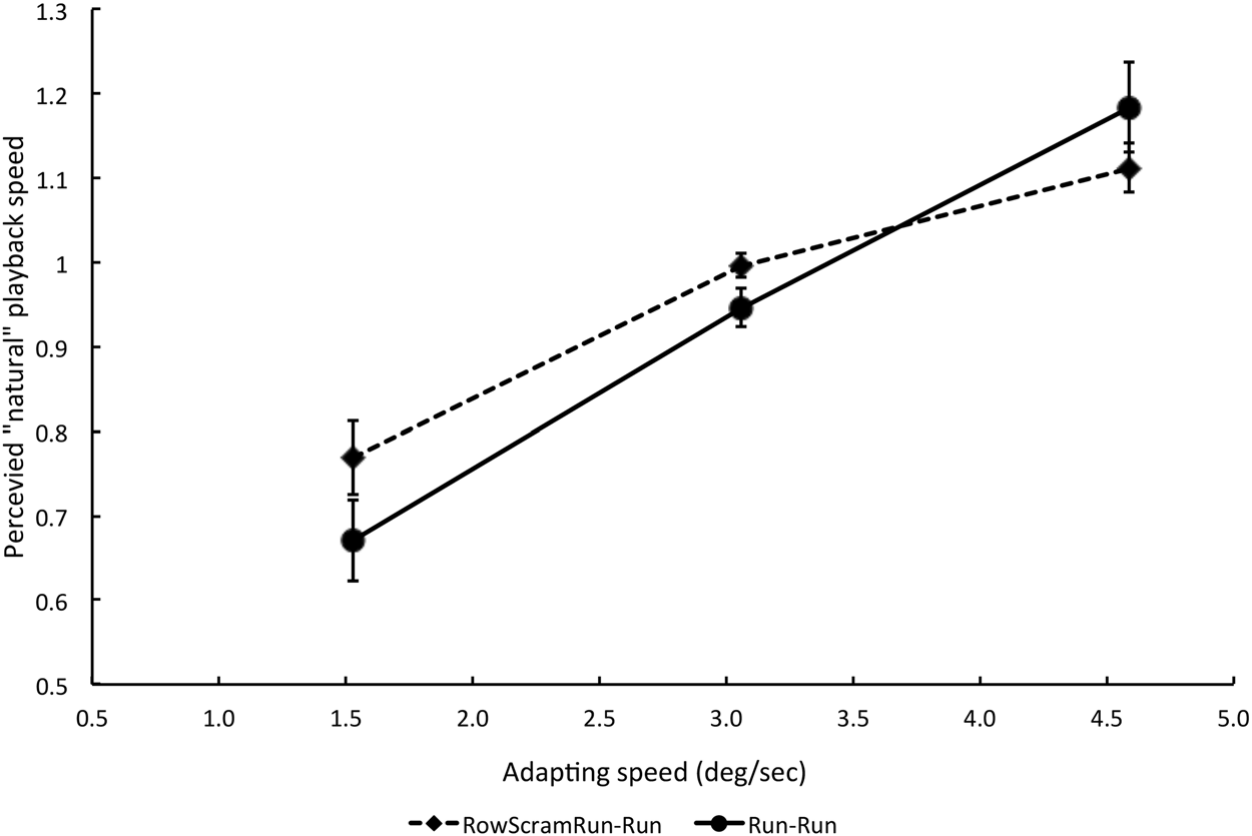
Results of Experiments 4 in comparison with Experiment 2, showing changes in apparently natural running speed using two different adapting patterns. *Filled circles* Data from Experiment 2 using running adapt and test videos. *Filled diamonds* Data from Experiment 4 using row-scrambled running adapt and running test videos. Figure convention as in Fig. 1. Each data point represents the mean P50 of five participants (+/−1 SE). An adapting speed of 3.06°/s corresponds to standard speed playback of running.

The results of Experiment 4 are shown by filled diamonds in Fig. 3. The effect of adapting speed using row-scrambled videos was highly significant (F (2, 8) = 22.79, p = 0.0001, n = 5). For comparison, the results of the intact Run-Run condition in Experiment 1 are also plotted. The same participants took part in the two experiments, and Repeated Measures Analysis of Variance revealed a significant main effect of adapting speed (F (2, 8) = 28.44, p = 0.0001, n = 5) but no significant main effect of scrambling (F (1, 4) = 3.546, p = 0.133, n = 5). The interaction was significant (F (2, 8) = 6.16, p = 0.024, n = 5), indicating a smaller effect of adaptation for scrambled videos compared to intact videos, which is evidence for a contribution from high-level form-based processes.

### Experiment 5: Retinotopic specificity of adaptation

The results of the previous two experiments are consistent with adaptation originating at lower levels of visual analysis where neural responses depend on retinal parameters. However the results obtained with scrambled videos indicate that relatively high-level processes are involved as well^9, 19^. We therefore conducted two experiments as further tests for the participation of high-level processes in the adaptation effect.

Previous research indicates that adaptation in high-level visual processes transfers to different retinal locations^19^, perhaps due to the involvement of large receptive fields in extrastriate cortex. Experiment 5 tested whether the adaptation found in previous experiments transfers to a different retinal location. In the previous experiments, adapting and test stimuli were always presented at the same visual location, and participants viewed them directly under free-viewing conditions (to simulate the natural conditions under which one usually views human locomotion). In Experiment 5 participants maintained fixation on a central red on-screen marker, and stimuli were presented either to the left or to the right of fixation. The same stimuli were used as in Experiment 2 (intact running adapt and test videos), but the retinal specificity of adaptation was tested by comparing results obtained when adapting and test stimuli were presented on the same side of fixation with results obtained when the adapting stimulus was presented on one side of fixation and test stimuli were presented on the opposite side of fixation. Brief same- and opposite-side test presentations were randomly interleaved within each experimental session to prevent participants from shifting their attention to a predictable test location in order to make their judgement in each trial.

**Figure 4:**
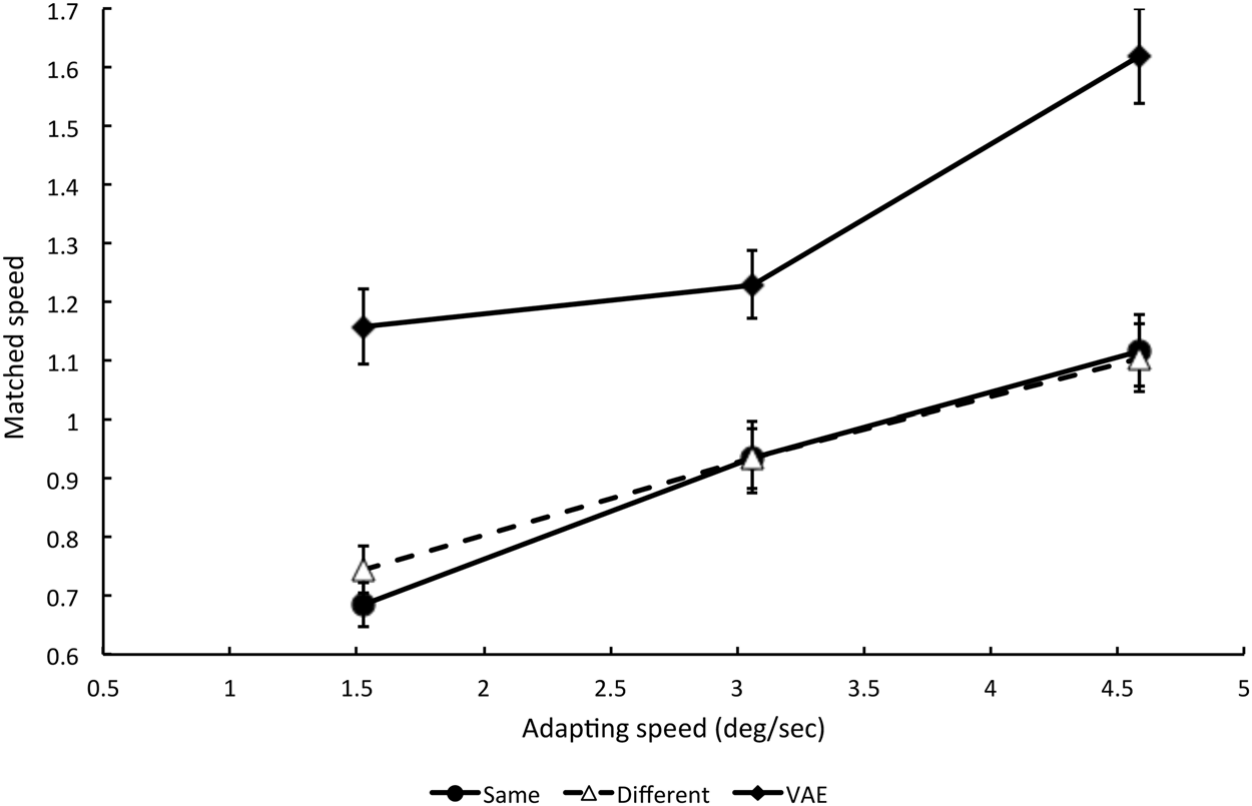
Results of Experiments 5 and 6 showing changes in apparent speed using two different stimulus paradigms. *Filled circles and unfilled triangles* Data from Experiment 5 to measure the retinotopic tuning of changes in apparently natural running speed, when adapting and test videos were presented at the same and different retinal locations respectively. Figure convention as in Figs 1 and 2. *Filled diamonds* Data from Experiment 6 to measure the velocity after-effect using row-scrambled running videos as adapting and test stimuli. Data points represent the speed of a test pattern at the adapted location relative to a comparison pattern at the unadapted location required to match a comparison stimulus moving at 3.06°/s (corresponding to standard speed playback of running). Values above 1.0 indicate that test pattern had to be presented at a higher speed than the comparison pattern in order to appear matched. Each data point represents the mean P50 of five participants (+/−1 SE).

The results of Experiment 5 are shown in Fig. 4. Results at the same and different retinal locations are shown by filled  circles and unfilled triangles respectively. Almost identical changes in apparent locomotion speed were obtained at the two retinal locations, indicating that the effect is not retinotopic. Repeated Measures Analysis of Variance confirmed a significant main effect of adapting speed (F (2, 8) = 102.59, p = 0.0001) but no significant effect of retinal location (F (1, 4) = 0.261, p = 0.636, n = 5). The interaction between speed and location was also not significant (F (2, 8) = 3.522, p = 0.08, n = 5).

### Experiment 6: Velocity after-effect

The lack of retinal specificity in apparent locomotion speed judgements contrasts with the specificity obtained in previous studies of adaptation to the speed of moving gratings (the velocity after-effect or VAE^20, 21^). A typical procedure in VAE studies is as follows. In the adapting phase a grating is presented (e.g. to the left of fixation), moving at a specific velocity. In the test phase two gratings are presented, one at the adapted location (e.g. left of fixation) and the other at an unadapted location (e.g. right of fixation). The two gratings move at different velocities, and the participant’s task is to report which grating appears to move faster. This technique is obviously designed to measure adaptation that is confined to the retinal area exposed to the adapting stimulus. A psychophysical comparison between stimuli presented at the unadapted location and test stimuli at the adapted location allows the experimenter to estimate changes in perceived speed caused by adaptation. VAE studies of adaptation to grating stimuli moving at similar retinal velocities to our stimuli (below 5°/s) typically show reductions in the apparent speed of all test stimuli following adaptation, rather than the symmetrical increases and decreases obtained in our experiments.

Thus the pattern of results of Experiment 5 in which we obtained similar adaptation effects at adapted and unadapted locations, is not consistent with an explanation of our effect in terms of low-level retinal velocity adaptation. However, it is possible that our stimulus design and placement somehow precluded the measurement of any retinotopic effect. To assess this possibility Experiment 6 tested whether our stimuli can generate a retinotopic VAE.

Both adapting and test stimuli were row-scrambled versions of running videos (identical to those used in Experiment 4), so no recognisable human forms were visible. Stimulus placement was identical to Experiment 5: Participants maintained fixation on a central red on-screen marker; the scrambled adapting stimulus was presented to the left of fixation, and test stimuli were presented on either side of the fixation marker. In each trial the playback speed of the two test stimuli differed, and the participant’s task was to report which scrambled test stimulus appeared to move faster. Adapting and test playback speeds were drawn from the same range of speeds as used in the previous experiments.

The resulting psychophysical functions allowed us to estimate the speed of the test stimulus presented at the adapted location required to match the apparent speed of the comparison stimulus at the other location. If the adaptation is retinotopic and consistent with previous VAE studies, then there should be a reduction in apparent test speed at the adapted location. If the adaptation is not retinotopic (or if the adaptation is ineffective) then there should be no measurable change in apparent test speed.

The diamonds in Fig. 4 show the results of Experiment 6, expressed in terms of the mean test speed which was required to match a comparison stimulus moving at 3.06°/s at the unadapted location (which corresponds to SS playback speed). All of the data points lie above 1.0, indicating that playback speed at the adapted location had to be higher than 1x in order to match SS playback at the unadapted location. The effect of adapting speed was highly significant according to a Repeated Measures Analysis of Variance (F (2, 8) = 22.53, p = 0.001, n = 5). Results are therefore consistent with previous studies of the VAE that show reductions in apparent retinal speed following adaptation, and different from those obtained in Experiments 1–5.

The results of Experiment 6 indicate that changes in apparent locomotion speed reported in the previous experiments are not consistent with an explanation in terms of a low-level VAE, for two reasons. First, adaptation-induced changes in apparent locomotion speed show both increases and decreases in apparent speed, whereas the VAE obtained using the same stimuli shows only reductions in apparent speed (in agreement with the literature). Second, adaptation to locomotion is not specific to retinal location, whereas the VAE is retinotopic. The implication of these results is that the two judgements engage qualitatively different visual processes; one is based on retinotopic information and the other is based on non-retinotopic information.

### Experiment 7: Adaptation to column-scrambled videos

Judgements of the relative speed of two meaningless visual stimuli are necessarily different from judgments of the ‘naturalness’ of human locomotion speed. The former judgement involves a direct comparison between two stimuli that are usually matched in all respects except their speed. The latter judgement involves a comparison between a single complex external stimulus and an internally maintained standard for locomotion speed. This fundamental difference must lie at the root of the different results obtained from VAE and locomotion speed experiments. Our experiments do show that changes in apparent locomotion speed depend on retinal stimulus values rather than norm values. What retinal properties could drive judgements of objective speed? One proposal in the velocity constancy literature^8–11^ is that image temporal frequency content (flicker) provides a cue to support judgements of real-world action speed. Temporal frequency is the product of a visual object’s retinal velocity and spatial frequency. So when the retinal spatial frequency of a moving object remains unchanged (in real-world terms, viewing distance is constant), temporal frequency increases as a function of the object’s speed. However, consider an object moving at a *fixed* real-world velocity: as viewing distance to the object increases, its retinal velocity decreases, but its retinal spatial frequency increases (projected size decreases). Consequently, the temporal frequency properties of the movement should remain stable, and therefore provide a cue as to the object’s real-world speed. Other things being equal, when an object’s visual motion involves a higher retinal temporal frequency then that object must be moving faster, whatever its viewing distance.

Experiment 7 tested whether changes in image temporal frequency content rather than velocity play a role in changes in apparent locomotion speed. We repeated Experiment 4, but made one important change to the adapting stimulus. In Experiment 4, coherent spatial form information was removed by randomly shuffling the order of the horizontal pixel rows in the adapting video. Each row of the re-ordered image sequence contained the same frame-to-frame displacements as the original video, thus preserving the predominantly horizontal motion signals generated by locomotion. In Experiment 7 the *columns* of pixels in the adapting video were randomly shuffled instead of the rows, with the same random order used in all frames of the video (see Supplementary Video V2 and Supplementary Figure F1). Column-shuffling preserved some information about spatial form, but destroyed horizontal motion signals by randomising sequential frame-to-frame luminance changes in horizontally adjacent pixels. However, the re-ordered video preserved the luminance modulation of each pixel created by the animation sequence; in order words, the temporal frequency content of the video. Different playback speeds necessarily altered these flicker rates because they altered frame duration and inter-frame interval.

**Figure 5:**
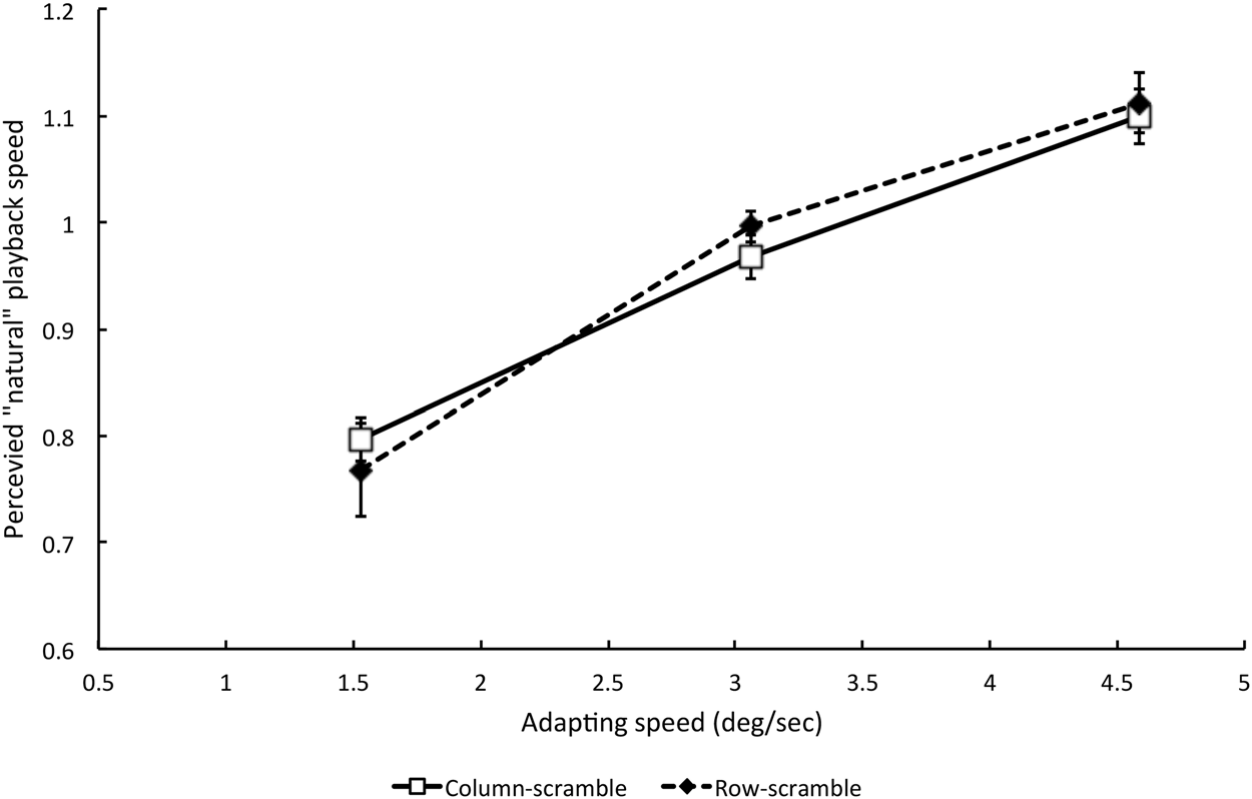
Results of Experiment 7 in comparison with Experiment 4, showing changes in apparently natural running speed after adaptation to scrambled running videos. *Filled diamonds* Data from Experiment 4 using row-scrambled adapt and test videos. *Unfilled squares* Data from Experiment 7 using column-scrambled adapt and test videos. Figure convention as in Figs 1 and 2. Each data point represents the mean P50 of five participants (+/−1 SE). An adapting speed of 3.06°/s corresponds to standard speed playback of running.

Results are shown in Fig. 5. For comparison, the results of Experiment 4 are also re-plotted from Fig. 2. Changes in the apparent speed of locomotion following adaptation were obtained even when no coherent horizontal motion was present in the adapting stimuli (unfilled squares), and the size of the effect was identical to that in Experiment 4 in which horizontal motion *was* present during adaptation (filled diamonds). A Two-Factor Mixed Analysis of Variance (different participants took part in the two experiments) confirmed that there was a significant main effect of adapting speed (F (2, 16) = 72.04, p = 0.0001, n = 10) but no significant difference between the two forms of scrambling (F (1, 8) = 0.036, p = 0.564, n = 10). The interaction between speed and scrambling was also not significant (F (2, 16) = 0.602, p = 0.56, n = 10). We conclude that the low-level component of adaptation is not driven by motion signals but by the flicker properties of the adapting stimulus. The effects of retinal velocity in Experiments 3 and 4 are therefore best characterised as effects of temporal frequency.

## Discussion

Experimental results show that adaptation-induced changes in apparent locomotion speed involve both low-level and high-level components. Our scrambling manipulations removed the local spatial correlations defining motion direction while preserving low-level temporal frequency content (see Supplementary Figures F1 and F2), and produced significant changes in apparent locomotion speed (see Figs 3 and 5). However the smaller adaptation effect with scrambled videos, and the lack of retinal specificity, point towards the involvement of high-level processes as well.

The changes in apparent locomotion speed we obtained in this series of experiments can be described as a form of perceptual re-normalisation with respect to an internally maintained norm for speed: Apparently natural speed was biased towards the locomotion speed to which participants were adapted, with no shift occurring after adaptation to natural speed itself (presumably because this speed matched the natural speed to which participants were already normalised prior to the experiment). Several other sensory attributes are thought to be represented relative to an internal norm that appears neutral or ‘normal’, including ‘white’ in colour perception, ‘static’ in motion perception, ‘sharp’ in image perception, and ethnicity in face perception^19, 22^. The position of the norm along the stimulus dimension is subject to bias by previous exposure to a stimulus lying on one side of the other of the norm value, shifting the norm towards the adapting value as in the effect we report. In the case of locomotion speed judgements, the norm or neutral point is the speed at which humans naturally move during locomotion, as determined by previous visual experience.

Re-normalisation should contribute to the maintenance of perceptual constancy because it ensures that the visual system continuously compensates for changes in the prevailing visual stimulation. Velocity re-normalisation may explain the common experience that, after driving at high speed in a de-restricted zone (120 km/h), vehicle speed is grossly underestimated when one enters a low-speed zone (50 km/h). In other words, high-speed driving re-normalises our perception of speed towards a high value, so that slower speeds subsequently appear even slower until re-normalisation occurs.

A range of different underlying neural processes have been proposed for achieving re-normalisation^19, 22^. In the case of colour perception, for example, re-normalisation seems to involve adjustments to the gain of a small number of low-level colour channels in order to match the statistics of the prevailing visual environment^22^. Our experiments indicate that re-normalisation of speed perception is driven by image temporal frequency content. Many previous studies of contrast sensitivity have found that the human visual system contains a relatively small number of channels tuned to different ranges of temporal frequency^23–26^; gain adjustments in these channels could provide a low-level component for re-normalisation of speed perception. Very few studies have tested for changes in perceived temporal frequency following adaptation to flicker, though one study has reported such an effect^27^.

A limitation of our experiments is that they were restricted to measurements of locomotion speed, but the underlying mechanism of re-normalisation should also operate for speed judgements of other objects. Indeed our results are consistent with previous research on velocity constancy using other image types^8–11^.

The adaptation effects obtained in our experiments could be due to changes in response or decision bias rather than to changes in visual processes^28, 29^. In some of the experiments, participants viewed stimuli which contained recognisable human forms, moving with the familiar appearance of slow-motion and fast-forward video playback. Participants were then asked to judge the speed of moving figures played at different speeds, so it would have been possible for participants to bias their responses by, for example, choosing to respond ‘faster’ more often after viewing slow-motion adapting stimuli^28^. However the results of Experiments 3, 4 and 7 are not consistent with bias accounts of the effect. They showed that the magnitude of the effect depends on the *retinal* properties of the adapting stimuli. It is well known that we do not ‘see’ the retinal image^30^ and find it extremely difficult to report retinal image properties^31, 32^; for example, the apparent speed of drifting gratings varies with viewing distance not retinal velocity^33^. Yet an explanation in terms of bias requires an assumption that participants were able to maintain an association between a specific degree of bias and a specific mean retinal velocity (or flicker rate) in the adapting stimulus, regardless of arguably more salient changes in other stimulus attributes.

The most persuasive argument against response or decision bias^34^ is that the effect can be clearly demonstrated using a video recording of any sports event such as a football game or a running race. If the video is viewed in slow-motion (or fast-forward) for about 30 seconds before being played at standard speed, an apparent increase or decrease in the speed of the action will be observed.

An implication of our results is that viewers can adapt or re-normalise to movies played back at slightly different rates, so that the action remains acceptably natural. Movie cameras and projectors in the silent film era were hand-cranked. Many movies appear to have been filmed at 16fps, but projected at rates ranging from 14 to 24fps for commercial reasons (performance scheduling^35^). Modern movies are recorded at 24 frames per second (fps) but when shown on PAL television (in Europe, China, Africa) their presentation rate is at 25fps. The acceptability of such variable projection rates to audiences may be helped by perceptual speed normalisation. Legal decisions based on reviewing slow-motion videos^17^ for signs of premeditation may also be influenced by re-normalisation.

In conclusion, a series of seven experiments found evidence for re-normalisation in judgements of the speed of a real-world action, human locomotion, which involves both low-level and high-level components. These studies represent the first using complex natural scenes to provide support for the temporal frequency theory of velocity constancy.

## Methods

### Ethics and Participants

All experiments were performed in accordance with institutional guidelines and regulations. The experimental protocol was approved by the School of Psychology Ethics Committee, University of Lincoln, UK. All participants gave their informed consent to take part in the experiments. Five participants took part in each experiment, drawn from a pool of seventeen volunteers (undergraduate and postgraduate students and staff at the university); four participants in each experiment were naïve, and one was an author (in all except Experiment 6, in which two authors participated). No single participant took part in more than four of the seven studies reported (modal participation was in two experiments). All participants had normal or corrected-to-normal vision.

### Apparatus

Stimuli were presented using a ViewPixx 3D Lite (VPixx, QC, Canada) flat panel display monitor, which has a spatial resolution of 1920 × 1080 pixels, a refresh rate of 120 Hz and screen width of 52 cm. The monitor was gamma-corrected using a LS100 luminance meter (Minolta, Osaka, Japan). A chin rest ensured that each participant viewed stimuli from a constant distance of 300 cm (0.0052° per pixel). Stimulus presentation and data collection were controlled by Psychtoolbox^36, 37^ for Matlab (The Mathworks Inc., Natick, MA), running on a Dell Windows PC.

### Stimulus Generation

Visual stimuli were prepared from two video recordings made using a high frame-rate camera (JVC GZ-GX1BEK, Yokohama, Japan). The video of running figures was recorded at the London Marathon in 2014; the video of walking figures was recorded on the High Street in Lincoln, UK in 2015. In both cases the camera was located on a tripod orthogonal to the path of the walking or running figures. In the Marathon video most of the figures moved from left to right, whereas in the High Street video right and left walking directions were approximately balanced. Recordings were made at 125 frames per second at a resolution of 720 × 576 pixels.

To prepare stimuli for use in the studies, individual frames were extracted from each video, converted to grey-scale and cropped to the central 512 × 512 pixels, yielding approximately twenty thousand frames per video. Each frame subtended 2.58° × 2.58° and was presented against a uniform mean luminance grey background (53.78 cd/m^2^).

Playback speed was controlled by two stimulus variables: frame duration (the number of 8.3ms screen refreshes for which each video frame was presented) and frame step (the offset between successively presented frames drawn from the original video sequence, namely every frame, every other frame, every third frame, and so on). Different combinations of frame duration and step were paired together to create the following available playback speeds relative to standard speed (1.0x): 0.48x, 0.58x, 0.64x, 0.72x, 0.80x, 0.96x, 1.12x, 1.20x, 1.28x, 1.34x, and 1.44x. The closest available speed to nominal standard 1x speed playback (frame duration set to one monitor refresh and frame step set to one frame) was actually 0.96x speed, due to the slight difference between the 125 Hz camera frame rate and the 120 Hz monitor refresh rate. Slow-motion adapting stimuli were played at 0.48x standard playback speed, and fast-forward adapting stimuli were played at 1.44x standard playback speed. These adapting speeds were used for all experiments to ensure comparability of results.

The accuracy of the video playback speed of our experimental stimuli was verified by applying the same processing workflow, presentation software and display equipment to a test video recording of time elapsed on an analogue clock face, made with the same video camera. Using the same combinations of frame duration and frame step, we verified with a stopwatch that the time elapsed on the clock face over a fixed period of several minutes agreed with that expected given the selected playback speed. To estimate the retinal speeds of individual figures visible during 0.96x playback of the videos, we counted the number of frames required for each of a sample of 63 figures to traverse the visible display. Given the refresh rate of the monitor and the angle it subtended at our viewing distance, it was straightforward to convert these frame counts to retinal speeds. Mean retinal speed in the walking video was 1.76°/s (SD = 0.98°/s), and in the running video was 3.06°/s (SD = 0.48°/s).

Row-scrambled videos were created by randomly shuffling the order of all pixel rows in a given video frame. The same shuffled order was used for all frames in a given presentation of an adapting or test video, in order to preserve the frame-to-frame horizontal displacements in each pixel row. However randomly different shuffled orders were used in different presentations. Column-scrambled videos were created by applying the same procedure to pixel columns in each video rather than pixel rows.

The space-time diagrams (*xt* and *yt* plots) in Supplementary Figure F1 and Fourier amplitude spectra in Supplementary Figure F2 show how row-scrambling and column-scrambling affected the information available in the videos. Row-scrambling preserved information about horizontal motion (spatiotemporal orientation) and luminance modulation (flicker), but destroyed information about spatial form. Column-scrambling retained some information about vertical spatial form (anorthoscopic form) and preserved flicker information, but destroyed information about horizontal motion.

### Procedure

All experimental procedures were approved by the School of Psychology Ethics Committee, University of Lincoln, UK. In each experiment the adapting video was shown for 30 s, followed by a repeating test/top-up cycle: After a brief 500ms interval containing a uniform grey field, a test clip appeared for between 475ms and 525ms (randomly selected, to avoid response cues based on a fixed duration or displacement distance), and was then replaced by a 500ms interval containing a uniform grey field. During this interval the participant pressed one of two available response buttons (ResponsePixx button box, VPixx, QC, Canada) to indicate their response. After a 500ms interval the adapting video re-appeared for a 5 s top-up of adaptation (this is a standard procedure for studies of motion adaptation). In all experiments a single experimental session involved adaptation to one playback speed (either 0.48x, 0.96, or 1.44x) and 140 test trials in which playback speed was drawn from seven possible values between 0.48x and 1.44x. Each test speed was presented 20 times in random order.

For most studies of locomotion speed judgements, adapting and test patterns were presented in the centre of the display under free-viewing conditions (no fixation mark or instructions) in order to more closely simulate judgements made under natural viewing conditions. To aid the participant in distinguishing between adapting clips and test clips, a 3–pixel blue border was drawn around the latter.

For Experiment 5 (test for retinotopic selectivity of adaptation), a small red fixation marker was provided at the centre of the display. The adapting stimulus was displaced to the left (near edge 0.26° from fixation); test stimuli could appear either at the same location or at the corresponding location to the right of fixation. Participants were instructed to maintain fixation on the marker at all times. Test location varied randomly between left and right locations from trial to trial in an experimental session, to avoid anticipatory eye movements. In all experiments involving judgements of locomotion speed the participant’s task was to decide whether the action in the test clip took place at a slower or faster pace than natural speed, and to press one of two available response keys to register their response.

The procedure for Experiment 6 (measuring the velocity after-effect using scrambled adapting and test patterns) was identical to that for studies of apparent locomotion speed, with the following exceptions. Stimulus locations matched those in Experiment 5: Participants fixated a small red central marker. The adapting pattern was displayed to the left of fixation (near edge 0.26° to the left of the fixation marker). The test display contained two videos, one to the left of fixation at the same location as the adapting stimulus, and the other to the right of fixation (near edge 0.26° away from the fixation marker). In each trial, one test video (standard) was played at 0.96x playback speed (mean velocity 3.06°/s), while the playback speed of the other test video (comparison) varied randomly from trial to trial over the same range of values as used in the locomotion studies. The task of the participant was to decide which of the two scrambled test clips, left or right, contained faster movement, and to press one of two available response keys to register their response.

### Data analysis

Each participant’s raw data from each adapting condition (20 responses at each of 7 test speeds) was collated to calculate the proportion of ‘faster’ responses as a function of test speed. A cumulative Gaussian psychometric function was fitted to the collated data using the Matlab LSQCURVEFIT() function which produced an optimal fit; the mean of the fitted function provided an estimate of the 50% point of participant’s responses. In studies of locomotion judgements, this point corresponded to the playback speed which the participant perceived as a natural pace; in the experiment on retinal velocity judgements the 50% point corresponded to the comparison speed which matched the apparent speed of the standard (which was played at standard-speed playback or 3.06°/s).

## Electronic supplementary material

Supplementary video V1

Supplementary video V2

Supplementary info
