# Supplementary material for: Visual adaptation alters the apparent speed of real-world actions
Source: Sci Rep. 2017 Jul 27;7:6738. doi: 10.1038/s41598-017-06841-5 (PMC5532221; doi:10.1038/s41598-017-06841-5)
Supplement: Supplementary file 3 — Supplementary info [file 41598_2017_6841_MOESM3_ESM.pdf]

Visual adaptation alters the apparent speed of real-world actions

George Mather, Rebecca J Sharman and Todd Parsons

Supplementary Information

Correspondence to gmather@lincoln.ac.uk

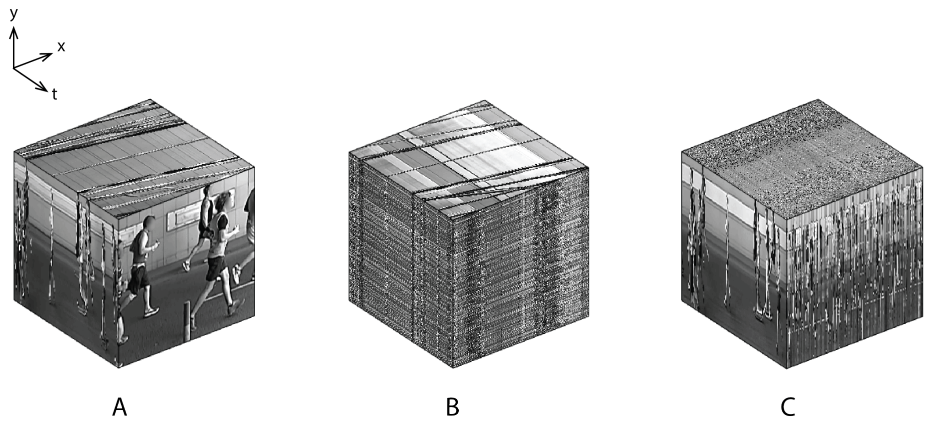

Supplementary Figure F1

Space-time diagrams created from animation sequences used in the experiments (faces have been blurred to prevent identification). The animations are represented as space-time volumes, each with dimensions *x*, *y*, and *t*. The *x* and *y* axes contain 512 pixels and the *t* axis contains 512 animation frames, creating a 512x512x512 cube.

Panel A Space-time volume created by intact running.

The right-hand vertical face of the cube shows the last frame (*xy* section) of the animation, depicting runners facing to the right.

The upper face of the cube shows a horizontal cross-section through the cube at a specific *y*-position, which creates an *xt* section; the rightward motion of each figure traces out a line through the *xt* section which slopes down towards the right (spatiotemporal orientation; different runners have slightly different speeds).

The left-hand vertical face of the cube shows the content of the left-most column of pixels in each animation frame as a function of time, which creates a *yt* section. Each running figure enters the animation at a specific frame, and takes several frames to pass through the column of pixels, rather like passing behind a very thin slit-shaped window placed in front of the animation, so ‘painting’ their profile across the time frames (anorthoscopic form; see Rock, I., 1981. Anorthoscopic perception. *Scientific American, 244*, 145-153).

It can be seen that the *xt* section carries information about the horizontal locomotion of the running figures, whereas the *xy* and *yt* sections carry information about the vertical form of the running figures.

Panel B Space-time volume created by row-scrambled running.

Row-scrambling shuffles the spatial order of horizontal rows of pixels in each animation frame, destroying the spatial information in the *xy* section (right-hand vertical face) which defines the form of the human figures. However, it does not interfere with the motion information in the *xt* section (upper face of the cube), because this section only shows pixel values within a given row. Row-scrambling also destroys the anorthoscopically defined forms in the *yt* section (left-hand vertical face), but preserves the temporal modulation of pixels in each row.

Panel C Space-time volume created by column-scrambled running.

Column scrambling shuffles the spatial order of vertical columns of pixels in each animation frame, destroying the spatial information in the *xy* section (right-hand vertical face) which defines the form of the human figures. It also destroys the coherent spatial displacements defining motion in the *xt* section (upper face) while preserving the temporal modulation of pixels in each column. Column-scrambling does not interfere with the anorthoscopic form information conveyed by the pixels within a given column (left-hand vertical face).

Results showed that intact adapting stimuli produced equivalent results to row-scrambled and column-scrambled stimuli, indicating that neither intact spatial form (only in A and C) nor coherent motion (only in A and B) were required to obtain the effect.

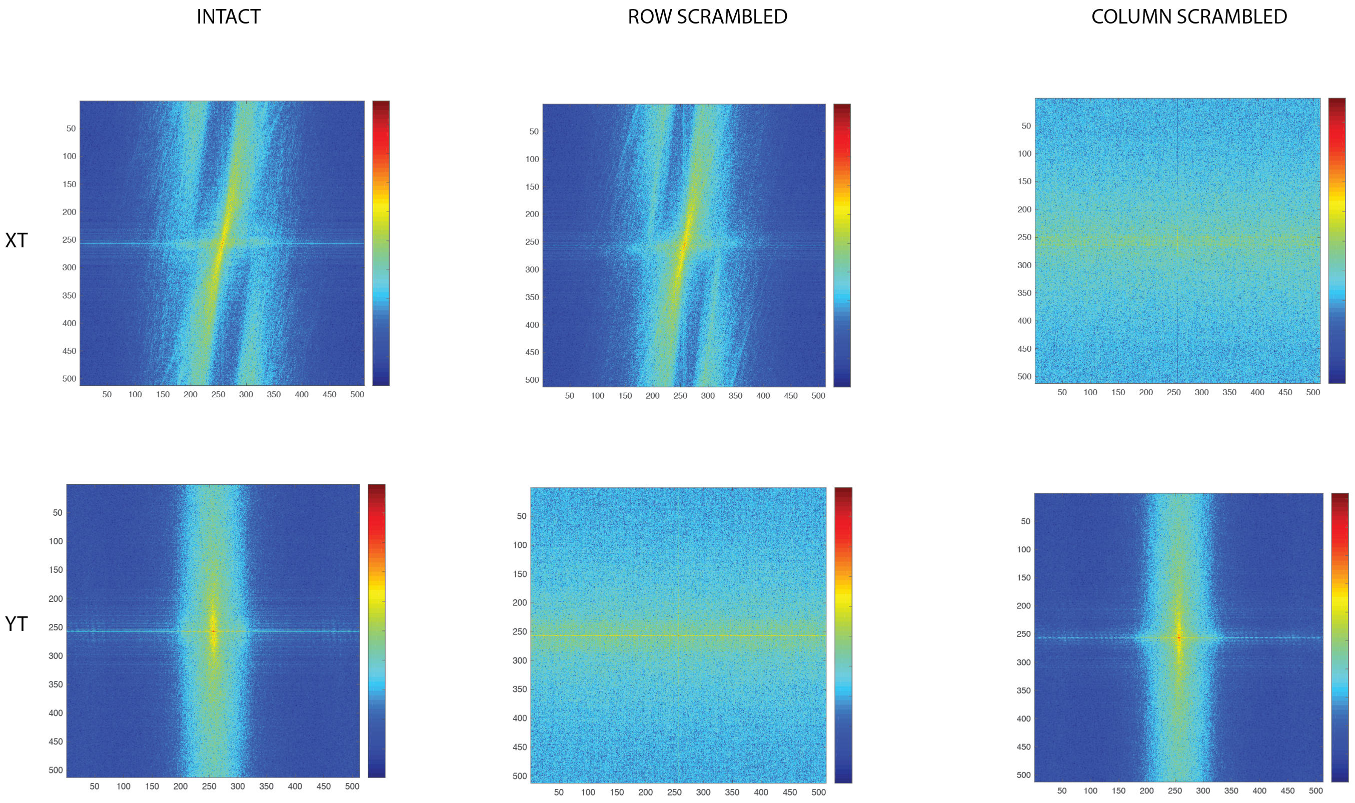
INTACT
ROW SCRAMBLED
COLUMN SCRAMBLED
XT
YT

Supplementary Figure F2

2D Fourier log amplitude spectra of *xt* and *yt* images as depicted in Supplementary Figure F1. Log amplitude is coded by colour, with the highest amplitudes in red/yellow and the lowest in blue. The upper row shows spectra of *xt* images in the three stimulus conditions (intact, row-scrambled, column-scrambled), and the lower row shows corresponding spectra of *yt* images. Zero frequency is at the centre of each spectrum, with spatial and temporal frequency represented on the *x* and *y* dimensions respectively. As in Figure F1, spectra were based on plots containing 512 pixels and 512 animation frames.

Intact videos (left-most spectra): The spectrum of the *xt* plot (upper spectrum) shows a 1/*f* decline in amplitude with increasing frequency in both spatial (*x*) and temporal (*y*) dimensions. The rightward horizontal motion of the figures creates oriented energy in the even quadrants of the spectrum, with slope corresponding to mean velocity. The spectrum of the *yt* plot (lower spectrum) also shows 1/*f* scaling in both dimensions but no oriented energy (because walking or running figures oscillate up and down).

Row-scrambled videos (middle spectra): The *xt* spectrum is intact because it is based on a single row in the scrambled video. The *yt* spectrum shows that spatial scrambling 'whitens' (flattens) the spatial frequency amplitude spectrum and removes spatiotemporal orientation (velocity) while preserving temporal frequency content (vertical modulation).

Column-scrambled videos (right-most spectra): Spatial scrambling randomises the *x*-position of pixels, so the *xt* spectrum (upper) is whitened in space. Velocity information is removed (no spatiotemporal orientation) but the temporal frequency content is preserved (vertical dimension in the spectrum). The *yt* spectrum (lower) is left intact in column-scrambled videos.

Note that temporal modulation is intact in all the spectra, but spatiotemporal orientation is preserved only in intact and row-scrambled videos.

Videos

Supplementary video V1.avi

A short video clip of the row-scrambled running stimulus, played at standard-speed.

Supplementary video V2.avi

A short video clip of the column-scrambled running stimulus, played at standard speed.
